# Hydrophobic ligands influence the structure, stability, and processing of the major cockroach allergen Bla g 1

**DOI:** 10.1038/s41598-019-54689-8

**Published:** 2019-12-04

**Authors:** Alexander C. Y. Foo, Peter M. Thompson, Lalith Perera, Simrat Arora, Eugene F. DeRose, Jason Williams, Geoffrey A. Mueller

**Affiliations:** 1 Genome Integrity and Structural Biology Laboratory, National Institute of Environmental Health Sciences, NIH, HHS, Research Triangle Park, NC 27709 North Carolina, USA; 2 Mass Spectrometry Research and Support Group, National Institute of Environmental Health Sciences, NIH, HHS, Research Triangle Park, NC 27709 North Carolina, USA

**Keywords:** Biochemistry, Structural biology

## Abstract

The cockroach allergen Bla g 1 forms a novel fold consisting of 12 amphipathic alpha-helices enclosing an exceptionally large hydrophobic cavity which was previously demonstrated to bind a variety of lipids. Since lipid-dependent immunoactivity is observed in numerous allergens, understanding the structural basis of this interaction could yield insights into the molecular determinants of allergenicity. Here, we report atomic modelling of Bla g 1 bound to both fatty-acid and phospholipids ligands, with 8 acyl chains suggested to represent full stoichiometric binding. This unusually high occupancy was verified experimentally, though both modelling and circular dichroism indicate that the general alpha-helical structure is maintained regardless of cargo loading. Fatty-acid cargoes significantly enhanced thermostability while inhibiting cleavage by cathepsin S, an endosomal protease essential for antigen processing and presentation; the latter of which was found to correlate to a decreased production of known T-cell epitopes. Both effects were strongly dependent on acyl chain length, with 18–20 carbons providing the maximal increase in melting temperature (~20 °C) while completely abolishing proteolysis. Diacyl chain cargoes provided similar enhancements to thermostability, but yielded reduced levels of proteolytic resistance. This study describes how the biophysical properties of Bla g 1 ligand binding and digestion may relate to antigen processing, with potential downstream implications for immunogenicity.

## Introduction

Due to their close association with human-built environments such as homes, schools and food service locations, the German cockroach (*Blatella germanica* or BGER) represents a major source of indoor airborne allergens. Indeed, studies on airborne dust samples obtained from urban schools and residences have identified the BGER allergens Bla g 1 or Bla g 2 in 26–66% of the samples tested^1,2^. In some inner-city environments where allergens were prevalent, an astounding 80% of individuals tested demonstrated sensitization to these allergens^2^. Additionally, BGER exposure and sensitization represents an important risk factor for the development of asthma, and has been correlated with an increase in emergency room visits and healthcare usage in response to the resulting respiratory symptoms^1,3–5^.

X-ray crystallography studies of the cockroach allergen Bla g 1 reveal a basic structural unit composed of two consecutive amino acid repeats consisting of 6 amphipathic alpha helices, which is unique in the protein database. Five of these helices arrange to form a planar pentagon while the 6^th^ is positioned above to form a hemispherical structure. Two consecutive hemispheres assemble to form the completed major allergy domain structure, which encloses an exceptionally large 3758 Å^3^ hydrophobic cavity at its center^6^. For context, a survey of around 600 enzymes produced a mean active site volume of 1072 Å^3^ ^7^. Hydrophobic cavities, with their reduced potential for hydrogen bonding, are even more restrained, with ~500 Å^3^ being proposed as a theoretical upper limit for most proteins; indeed, a survey of several non-specific lipid transfer proteins (nLTP’s) reveals cavities in the 100 Å^3^–400 Å^3^ range, consistent with this limit^8,9^. Previous studies show that Bla g 1 is able to bind a range of phospholipids and fatty acids, and the available X-ray structure of Bla g 1 reports electron density within the central cavity suggesting it may act as a lipid -binding site. However, the exact mode of binding and its contribution to Bla g 1 structure, stability, and allergenicity remains unknown.

The distribution of allergens across the sequence space is biased in that allergens are found in only 2% of all known protein families^10^. This suggests the existence of specific structural and/or functional characteristics that allow certain proteins but not others to induce allergic sensitization. Understanding these characteristics remains a key goal in allergology. A survey of the allergen database suggests that numerous allergens are expected to bind lipids and other hydrophobic molecules^10^, potentially representing an important molecular determinant of allergenicity for proteins like Bla g 1. These ligands can act as potent stimulators of TLR2, TLR4, and CD1 signalling pathways that modulate the immune response generated against any accompanying proteins^10–13^. Indeed, bacterial lipids such as LTA and LPS are commonly found in dust particles, where they have been shown to enhance the immune response against co-administered indoor and pollen-based allergic proteins^14–18^. Similar effects have been observed for other pollen and food-derived lipids^19–21^. Thus, allergen-associated lipids can potentially act as adjuvants, enhancing the efficacy of the former through their innate stimulatory properties.

Another possibility is that binding of hydrophobic ligands into enclosed cavities such as that found in Bla g 1 could alter the allergens’ structural or biophysical properties in a manner that promotes immunogenicity, providing an additional mechanism through which lipids can potentially contribute to the sensitization process. One area which may be sensitive to such changes in allergen structure or stability is its degradation and subsequent processing within the endosomal compartment of antigen-presenting cells. Here, proteases such as cathepsin S cleave the allergen to produce peptide fragments, which are loaded onto the type two major histocompatibility complex (MHCII) and exposed on the surface for T-cell recognition. Since MHCII loading occurs late in the endosomal lifecycle, the rate at which this processing occurs may influence immunogenicity, as only antigens and epitopes which are able to persist into the late endosomal compartment will generate an immune response^22–27^. Since proteases typically act on unstructured sequences^22,28^, differences in endosomal processing rates provides a potential mechanism through which the innate biophysical properties of an allergen can influence immunogenicity. This is best exemplified by a series of recent studies in which isoforms of Bet v 1 that display enhanced stability are able to avoid premature processing by cathepsin S, yielding a stronger allergic (T_H_2) response than their less allergenic variants^23,24^. It is possible that binding of lipid cargoes into Bla g 1 could elicit a similar response; modulating allergen stability and endosomal processing in a manner which enhances sensitization. This article explores this hypothesis using molecular dynamics simulations coupled with *in vitro* biophysical assays and simulated endolysosomal degradation reactions to characterize the structural basis for lipid binding to this unique protein fold, and its implications for Bla g 1 structure, stability, and processing.

## Results

### Purification of Apo-Bla g 1

**Figure 1:**
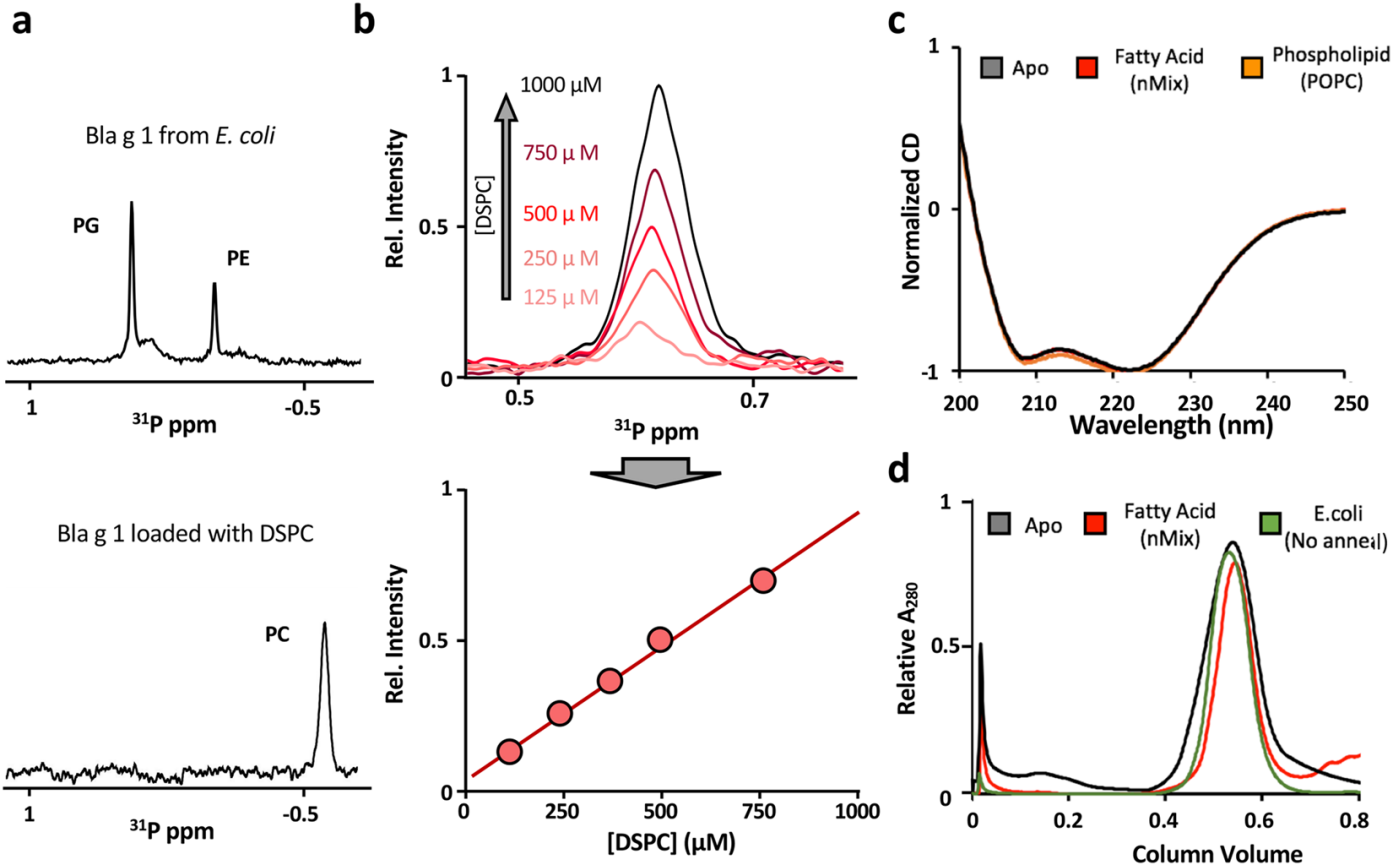
Comparison of Apo and Cargo-loaded Bla g 1. ^31^P-NMR spectra (**a**) of Bla g 1 purified directly from E. coli (top), and DSPC-loaded Bla g 1 purified using reverse-phase HPLC followed by the annealing protocol (bottom). The DSPC standard curve used to estimate Bla g 1 binding stoichiometry from ^31^P-NMR intensities is shown in (**b**). CD spectra (**c**) obtained for Bla g 1 purified using reverse-phase HPLC coupled with annealing in the absence of cargo (Black). Spectra of Bla g 1 loaded with nMix, a mixture of palmitate, oleate, and stearate fatty acids which mimics the cargo identified from Bla g 1 obtained from its natural allergen (Red), or 1-palmitoyl-2-oleoyl-phosphatidylcholine (POPC) phospholipid (Orange) cargo are shown for comparison. Representative gel-filtration elution profiles (**d**) for Apo (Black) and nMix-loaded (Red) Bla g 1 generated through the annealing process, and Bla g 1 purified from E. coli without annealing (Green).

Previous studies demonstrated the ability of Bla g 1.0101 (34–216, hereafter referred to as Bla g 1) to bind a heterogeneous range of lipids and hydrophobic cargoes, which remain associated throughout the purification process^6^. While these retained lipids provided valuable insight into the phospholipid headgroup preferences of Bla g 1, their heterogeneous nature precluded any systematic study of lipid-allergen interactions. In order to remove the endogenously-bound lipids from the *E. coli* expression system, Bla g 1 was further purified using a reverse-phase C18 column (see details in methods section). The resulting protein was then lyophilized and annealed in aqueous buffer to produce apo-Bla g 1. To generate samples of Bla g 1 that were uniformly loaded with a defined cargo the annealing process was carried out in the presence of >16:1 molar excess of the desired molecules. ^31^P NMR spectra obtained for Bla g 1 loaded with distearoyl phosphatidylcholine (DSPC) lipid in this manner show the presence of the desired phosphatidylcholine (PC) cargo while no peaks from the recombinant expression system (phosphotidylethanolamine, PE, and phosphotidylglycerol, PG, lipids) are detected, suggesting that the cleaning and loading protocol was successful (Fig. 1a); a conclusion supported by thin layer chromatography studies coupled with iodine staining, or CuSO_4_-phosphoric acid charring (Supplementary Fig. S1). Comparing the peak intensity of the DSPC-loaded Bla g 1 against a standard curve of known concentrations of DSPC yields a binding stoichiometry of 4.7 ± 0.5 phospholipids per molecule of Bla g 1 respectively (Fig. 1b). Circular dichroism (CD) spectra obtained for Bla g 1 prepared using this method displays the characteristic minima at ~208 and 222 nm indicative of an alpha-helical protein consistent with the available crystal structure^6^, suggesting that the structure of Bla g 1 is recovered after the purification and annealing process (Fig. 1c). Finally, gel-filtration chromatography of apo and cargo-loaded Bla g 1 yields elution volumes similar to those obtained from Bla g 1 purified directly from *E. coli* (Fig. 1d), indicating that the annealing and lipid-loading process does not result in the formation of aggregates or other higher-order lipid-allergen structures

### Cargo binding introduced minimal perturbations to Bla g 1 structure

**Figure 2:**
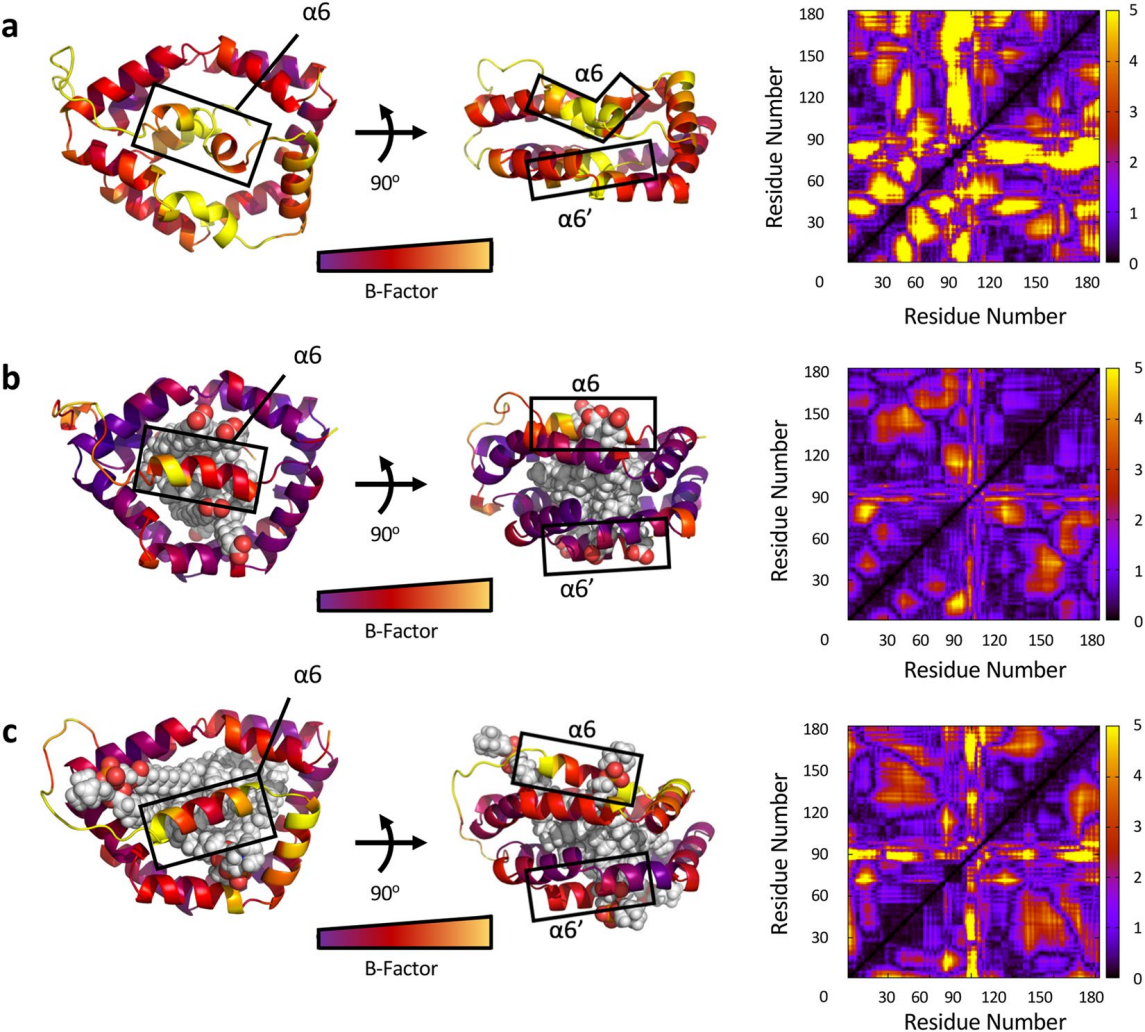
Molecular Dynamics Simulations of Ligand Binding to Bla g 1. Structural models of Apo-Bla g 1 (**a**), Bla g 1 loaded with 4 phosphatidylcholine phospholipid cargo (**b**), or with eight saturated C18 fatty acids (**c**) obtained through 120 ns molecular dynamics simulation. Structures colour-coded by B-factor values. Cα displacement values for all residues averaged over the last 30 ns of the simulation are shown on the right.

To determine whether the structure of Bla g 1 is perturbed in a more subtle manner than is appreciable from the above techniques, molecular dynamics calculations were carried out. A structural atomic model was created for Apo-Bla g 1 using the previously solved X-ray structure as a template, and allowed to equilibrate for a total of 120 ns (Fig. 2a). The available crystal structure for Bla g 1 described electron density within the central hydrophobic cavity which resembled that of a bound phospholipid^6^. Using this information, along with the binding stoichiometry obtained through ^31^P-NMR, an additional model was constructed depicting Bla g 1 bound to its likely full complement of 4 phospholipids identified in the previous section, for a total of 8 acyl chains (Fig. 2b). Given the headgroup preference observed in Bla g 1 purified from *P. pastoris*, a saturated C18 phosphatidylcholine phospholipid was chosen for these studies^6^. In the same study, Mueller *et al*. identified the endogenous cargo of natural Bla g 1 purified from frass (nBla g 1) as being a mixture of C16-C18 fatty acids. To best replicate these conditions, an additional model was created with Bla g 1 bound to eight saturated 18-carbon fatty acids (stearate) again likely representing full stoichiometric binding (Fig. 2c). In both cases the C18 acyl chains are able to completely fill the interior of the hydrophobic cavity with little void volume remaining, and minimal structural perturbations compared to the initial X-ray structure, consistent with the experimentally determined stoichiometry.

A comparison of the resulting Apo, phospholipid-loaded and fatty acid-loaded structures shows changes in the position and conformation of helices 6 and 6’. In the unloaded Bla g 1 these helices collapse into the cavity to fill the interior cavity. Binding of fatty acid or phospholipid cargoes causes this cavity to be occupied by upwards of 8 acyl chains, allowing 6 and 6’ to expand outwards, mirroring their conformation depicted in the available X-ray structure. Here helix 6 and 6’ divide the exterior face of the central cavity, forming four semi-circular openings (two per face) which are able to accommodate two acyl chains each, representing either two fatty acid ligands or a single phospholipid each. In the case of the former, the two fatty acid headgroups are able to effectively shield the occupied hydrophobic cavity from the aqueous environment. In contrast, the single headgroup of phospholipid ligands is unable to carry out this task effectively, necessitating perturbations to helix 6/6’ and the downstream loop region in order to minimize unfavourable interactions between the hydrophobic central cavity and the outside environment, contributing to the elevated dynamics observed for this region in the diacyl-chain bound model (Fig. 2). Despite these differences, the pentagonal rings formed from helices 1–5 and 1′–5′ are generally well aligned in all three models, conserving the overall α-helical structure of Bla g 1 (Fig. 2). This conclusion is further supported by circular dichroism spectra obtained for Bla g 1 loaded with a range of lipid and fatty acid cargoes of varying acyl chain lengths which showed no significant change in secondary structure (Fig. 1c)

### Fatty-acid and phospholipid binding reduces Bla g 1 conformational dynamics

To explore the consequences of ligand binding on Bla g 1 dynamics, molecular dynamics (MD) simulations were run on the apo and cargo-loaded atomic models over a 120 ns window, from which the temperature values (B-factor) and root mean square fluctuations in Cα position for each residue were obtained (Fig. 2). A high degree of conformational dynamics was observed for the apo structure, with the majority of the fluctuations localized to the hemispherical subunit corresponding to the N-terminal portion of the Bla g 1 sequence. Binding of both fatty acid and phospholipid cargoes significantly reduced the structural fluctuations of Bla g 1. While the former significantly reduced dynamics across the entire structure, significant dynamics remain in the 6’ and linker region when loaded with the latter, potentially reflecting the localized perturbations required to accommodate diacyl chain ligands as described in the previous section.

**Figure 3:**
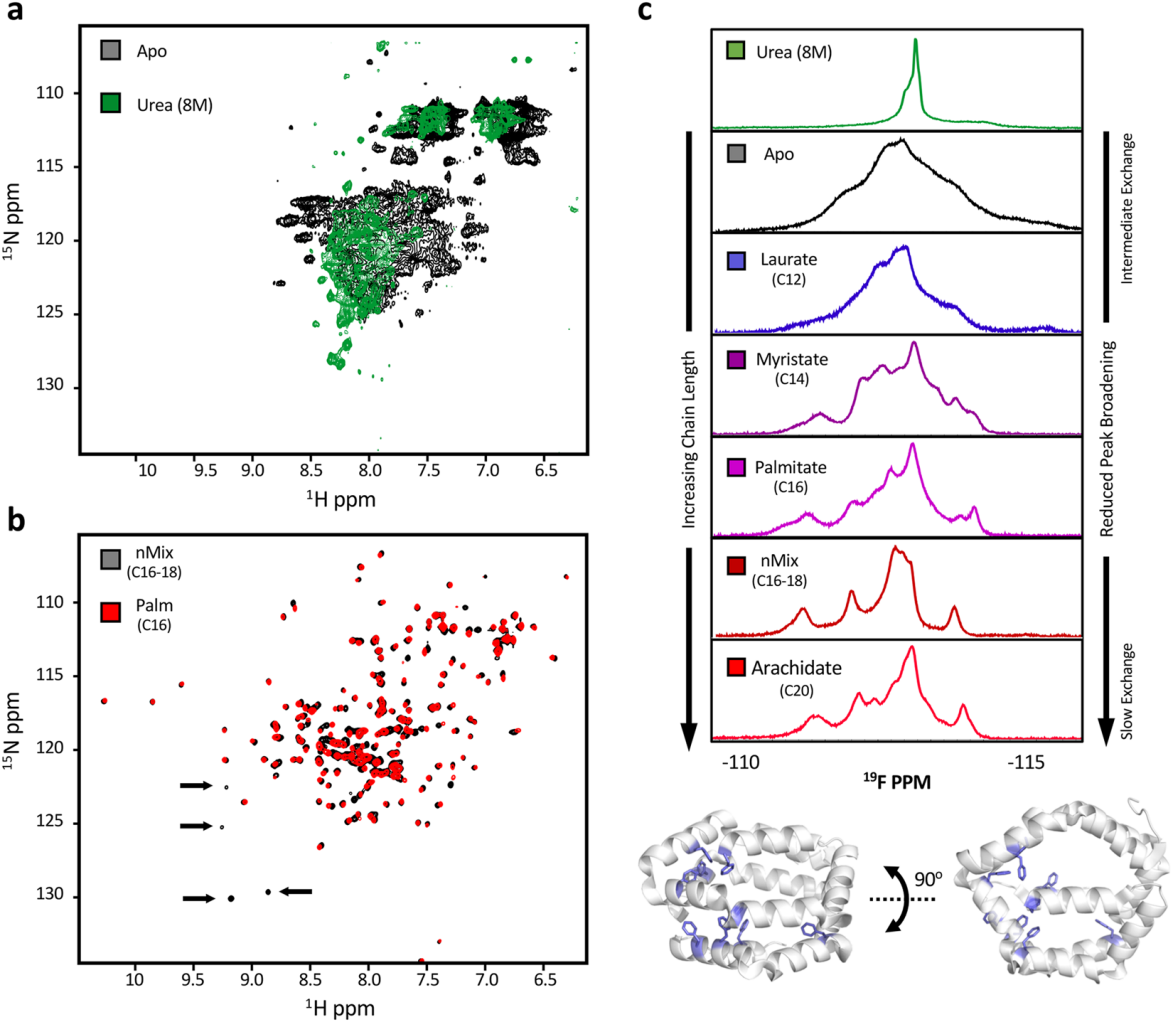
Examining Bla g 1 Dynamics Using NMR. ^1^H-^15^N-NMR spectra of Apo-Bla g 1 in the absence (Black) or presence (Green) of 8 M urea (**a**) showing significant peak broadening indicative of intermediate (μs-ms) exchange dynamics. Spectra of Bla g 1 loaded with nMix (Black) or palmitate (Red) fatty-acid cargoes is shown in (**b**). Arrows indicate some of the peaks which experience broadening in the C16 sample relative to nMix. ^19^F-NMR spectra obtained for 3-fluorophenylalanine-labeled Bla g 1 in the absence and presence of various fatty-acid cargoes (**c**) showing the dependence on peak broadening on cargo acyl chain length. Spectra of denatured Bla g 1 in 8 M urea is shown for comparison (Green). Structure of Bla g 1 illustrating the location of all phenylalanine residues (Blue) is shown at bottom. PDB ID: 4JRB

NMR is a technique that can be used to report on the conformational dynamics of proteins making it an empirical compliment to the computational studies described above. Typical ^15^N protein labelling of Apo-Bla g 1 gave rise to poor quality spectra with extensive peak broadening which did not allow for unambiguous assignments (Fig. 3a). Addition of 8 M urea resulted in a further reduction of peak dispersion in the proton dimension and peak narrowing, suggesting that the poor quality of the Apo-Bla g 1 spectra is a result of extremely unfavourable backbone conformational dynamics on the intermediate (μs-ms) timescale rather than protein unfolding; a conclusion supported by the previously presented circular dichroism and atomic modelling data. Binding of nMix (C16-C18) fatty acid cargoes reduced broadening (Fig. 3b), indicating a change in conformational exchange rates, which may reflect the structural fluctuations obtained from the atomic models described above. Similarly, ^19^F-NMR spectra of 3-fluoro-phenylalanine labelled apo-Bla g 1 show extensive broadening of the fluorophenylalanine side-chain peaks (Fig. 3c), the dispersion of which was notably reduced by the addition of 8 M urea, yielding a single sharp peak in the middle of the spectrum. As with the ^1^H-^15^N spectrum, addition of fatty acid significantly reduced peak broadening while enhancing dispersion (Fig. 3c), with both effects showing a strong dependence on alkyl chain length suggesting that different cargoes might give rise to varying levels of structural stabilization. This hypothesis is supported by the ^1^H-^15^N spectra, in which some peaks show moderate broadening in the palmitate (C16)-loaded sample relative to its nMix (C16-C18) counterpart (Fig. 3b)

### Reduced Bla g 1 dynamics correlate with enhanced thermostability

**Figure 4:**
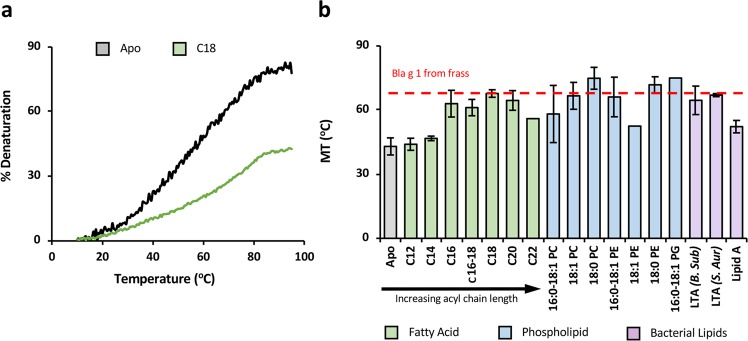
Influence of Hydrophobic Cargo Binding on Bla g 1 Thermostability. Representative thermal melt curves (**a**) tracking the relative loss of CD signal intensity at 220 nm for apo-Bla g 1 (Black) at increasing temperatures. The curve for Bla g 1 loaded with a representative fatty acid (C18 stearate) cargo is shown in green. Note that the C18-loaded sample does not reach 50% denaturation. As such, the melting temperature (T_M_) as employed throughout this manuscript is defined as the temperature at which 25% denaturation is observed. T_M_ values for Bla g 1 in the presence of various fatty-acid (C12–C22) and diacyl-chain (C16–C18) cargoes are shown in (**b**). The T_M_ for Bla g 1 obtained from its natural allergen source is shown in red for reference. The error for this assay and all subsequent data presented in this work represent the standard deviation about the mean obtained for at least three independent trials representing at least two biological replicates unless otherwise specified. Raw data shown in Supplementary Table S2a.

The observed reduction in conformational flexibility could potentially correlate with an increase in thermostability due to more favourable enthalpic interactions. To explore this hypothesis, thermal denaturation experiments were carried out on apo and cargo-loaded Bla g 1, using the CD signal at 222 nm to monitor unfolding as described previously^29^. As shown in Fig. 4, binding of fatty acids resulted in a notable increase in melting temperature (T_m_), the magnitude of which is dependent on the ligand acyl chain length consistent with the ^19^F NMR studies with maximum stabilization (~20 °C) observed with C16–C18 ligands, mirroring the preference for palmitate, oleate and stearate (C16:0, C18:1, C18:0) fatty acid cargoes observed in nBla g 1. This high level of stabilization was experimentally verified in nBla g 1 and could be replicated by loading recombinant Bla g 1 loaded with an equimolar mix of all three fatty acids (nMix).

Binding of 16 and 18-carbon PG, PE, and PC phospholipids yielded similar enhancements as their fatty acid counterparts. However, lipid A, the lipophilic portion of the bacterial outer membrane lipopolysaccharide (LPS), failed to yield a notable increase in stability. This lack of binding was further confirmed through the *Limulus* Amebocyte Lysate (LAL) assay which revealed a binding stoichiometry of ~1.2 × 10^−3^ for the lipid A-treated allergen, with similar values being reported for both the Apo and nMix-loaded Bla g 1. This lack of binding suggests that the opening into the Bla g 1 hydrophobic cavity is unable to accommodate the greater number of acyl chains, likely due to the position of capping helices 6 or 6’. In line with this hypothesis, Bla g 1 could be loaded with lipoteichoic acid (LTA); another bacterial cell wall component containing two acyl chains with large (~5–7 kDa) and varied glycerophosphate and glycopeptide subunits^30^. LTA’s from both *B. subtilis* and. *S. auerus* yielded similar enhancements in thermostability as their equivalent 14–16 C phospholipids and fatty acids^30,31^. The ability of such a wide range of ligands to enhance Bla g 1 thermostability suggests that this interaction is relatively nonspecific, with binding site accessibility being the main limitation.

### Enhanced Bla g 1 thermostability hinders processing by endosomal proteases

**Figure 5:**
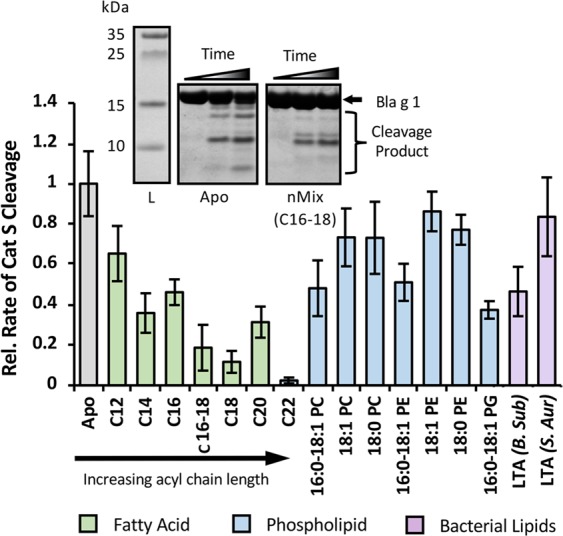
Influence of Hydrophobic Cargo Binding on Endosomal Proteolysis. Proteolysis rates of cargo-loaded loaded Bla g 1 by cathepsin S relative to Apo-Bla g 1. Proteolysis of fatty-acid loaded Bla g 1 (Green) is dependent on acyl chain length. Diacyl chain (Blue) and LTA (Purple) cargoes yield a more modest response. SDS-PAGE of the peptides produced from the cleavage of apo and fatty acid (mix) loaded Bla g 1 (inset) suggests that cargo binding alters both the rate of proteolysis, and the fragments generated. Raw data shown in Supplementary Table S2b. Full-length image of SDS-PAGE gels shown in Figs. S4 and S5.

Proteolytic cleavage of potential allergens by endosomal proteases, particularly cathepsin S (Cat S) is required for the generation of T-cell epitopes^32^. To explore the possibility that Cat S cleavage of Bla g 1 is influenced by lipid cargo binding, proteolysis rates were measured for both apo and cargo-loaded Bla g 1 under endosomal conditions^23^. SDS-PAGE analysis revealed a linear correlation between Cat S proteolysis and Bla g 1 concentration for all conditions tested, suggesting that all measurements were obtained within the linear range, and that any differences in proteolysis rates between the various conditions tested represent variations in catalytic turnover (k_cat_) rather than binding affinities (K_D_ or K_m_). Samples of Bla g 1 loaded with fatty acids showed a chain length-dependent decrease in the rate of proteolytic cleavage, which broadly anticorrelates with thermostability (Fig. 5). Addition of fatty acids after thermal annealing of the protein to the reaction did not alter the rate of proteolysis (Supplementary Fig. S3), suggesting that the reduced proteolysis reflects the stabilizing effect of the ligand, rather than any inhibitory effect which might be exerted by residual, free fatty acid micelles.

**Figure 6:**
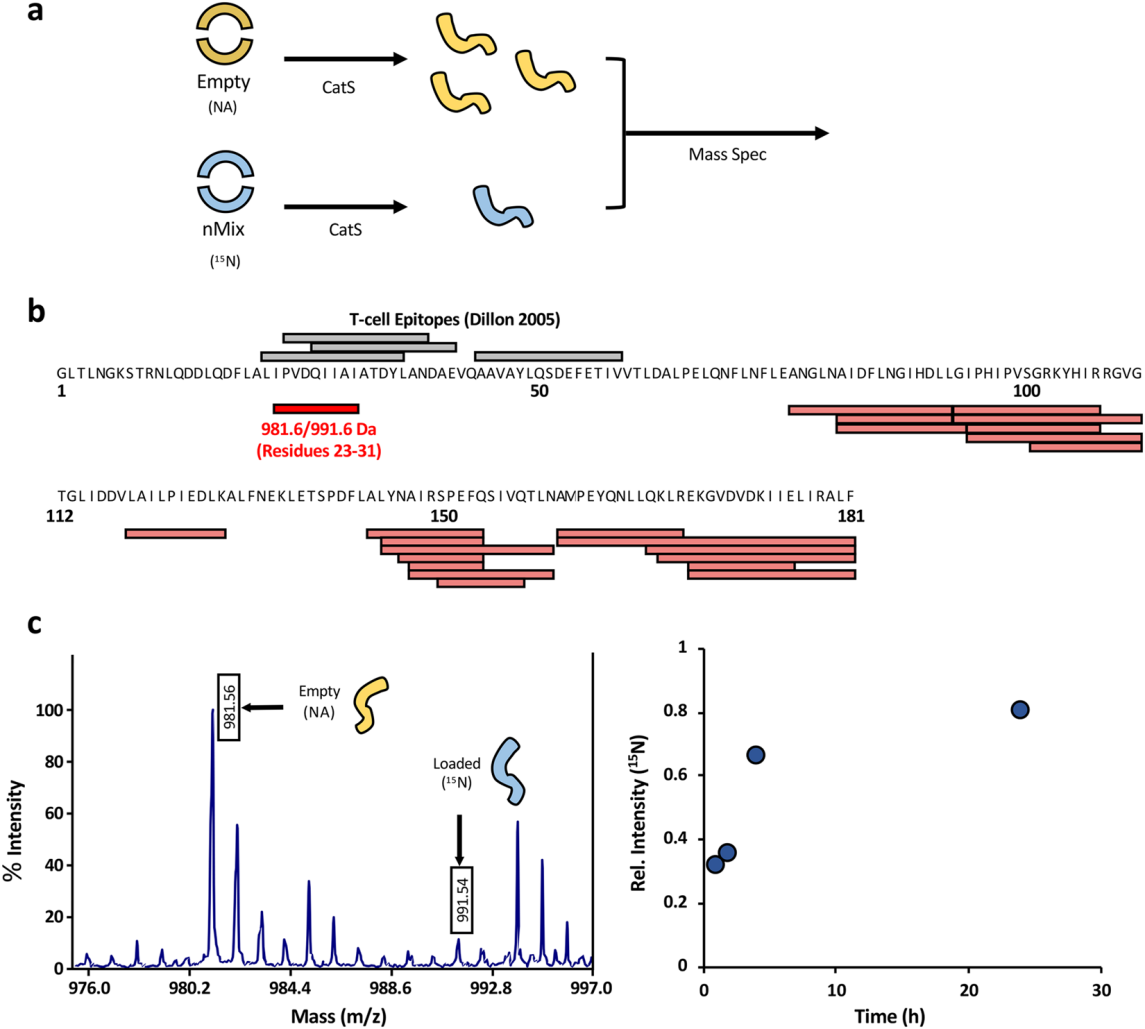
Assessing Cat S Proteolysis and T-cell Epitope Generation via Quantitative Mass Spec. The overall scheme describing the approach to quantitative mass spec analysis of epitope generation is shown in (**a**). Samples of empty natural abundance (NA) and cargo-loaded uniformly ^15^N-labeled Bla g 1 are generated and subjected to Cat S proteolysis. Equal amounts of sample from each reaction are combined and run simultaneously using MALDI. The mass difference between the natural abundance and ^15^N-labeled samples allows differentiation between fragments produced from the cleavage of the empty and loaded Bla g 1. Since both samples were prepared under identical conditions and run simultaneously, the relative peak intensities reflect the relative amounts of fragments generated during the Cat S cleavage step. (**b**) shows the peptide fragments observed after a 4 h cathepsin digestion mapped onto the Bla g 1 sequence (Red) compared to the known T-cell epitopes identified by Dillon *et al*. (Black). A detailed view of the MALDI peaks corresponding to the 981.6/991.6 Da epitope fragment after a 4 h digestion is shown in (**c**). The ratio of ^14^N:^15^N peak intensities for this peptide over a 24 h time-course is shown on the right.

To assess the effect of cargo loading on the generation of specific peptide fragments, cathepsin S digest reactions were carried out on natural abundance (NA) apo and cargo loaded, uniformly ^15^N-labeled Bla g 1. The resulting products were then pooled and analysed simultaneously using MALDI-MS, allowing us to measure the relative abundance of the ^15^N and NA cleavage fragments, and thus the relative rates of proteolysis in a quantitative manner (Fig. 6a). From the MALDI spectrum of the 4 h time point, about 20 peptides could be readily assigned and observed as isotope pairs, and further 6 NA peptides were identified without a corresponding ^15^N counterpart (Fig. 6b, Supplementary Fig. 3). The relative abundance of all ^15^N cathepsin S fragments is significantly lower (20–40%) than their NA counterpart, confirming the reduction in proteolysis observed previously (Supplementary Table S5). A number of these fragments correspond to cleavage sites within the region encompassing helix 5 and 6, along with the flexible intra-domain linker region (residues 66–100). While this region is stabilized by the binding of fatty acid ligands, the localized perturbations required to accommodate diacyl chain cargoes may leave it susceptible to proteolysis, accounting for the reduced proteolytic resistance observed for phospholipid and LTA ligands relative to their fatty-acid counterparts. Another fragment that was differentially detected in the apo and nMix-loaded reactions could be mapped to Bla g 1 residues 23–31 (m/z^14^N = 981.6, m/z^15^N = 991.6 Da). This region overlaps with several known T-cell epitopes identified by Dillon *et al*.^33^ (Fig. 6b), providing a possible link between ligand binding and immunogenicity^33^. To further evaluate the effect of Bla g 1 cargo loading on the formation of this peptide, the ratio of the m/z 991.6 ion to the m/z 981.6 ion was evaluated over a more extensive time course. At earlier timepoints, the peak intensity of the ^15^N (loaded) version of this peptide is significantly reduced relative to its ^14^N counterpart (Fig. 6c). This difference is somewhat reduced at later timepoints, likely due to the NA (empty) cathepsin S cleavage reaction reaching completion. Nonetheless, a small but notable difference remains even after 24 h. This provides direct evidence that cargo loading inhibits T-cell epitope fragment generation of the ^15^N-labeled sample under simulated endosomal conditions employed here and elsewhere in the literature^23^, further supporting a link between Bla g 1 ligand binding, endosomal proteolysis, and epitope generation.

## Discussion

Lipid binding has been proposed to represent a functional property of many allergenic proteins, with many allergens including albumins, lipocalins, non-specific lipid transfer proteins (nLTP) and globulins displaying an ability to bind hydrophobic ligands within an internal binding site. However, to the best of our knowledge none are able to match the sheer size and binding capacity of the Bla g 1 hydrophobic cavity described in this study. This hydrophobic cavity provides an uninterrupted, spherical 3758 Å^3^ space which we show here may be occupied by up to 8 ligand molecules. For comparison, most other lipid-binding allergen families typically enclose a single 100 Å^3^–400 Å^3^ cavity that accommodates a binding stoichiometry ≤1^9,34,35^. Two notable exceptions are serum albumins, and the group 5 dust mite allergens Der p 5. The former employs multiple binding sites allowing for a theoretical binding stoichiometry approaching that of Bla g 1. However, studies on the human albumins reveal that only 0.5–2 of these sites are occupied under physiological conditions suggesting that lipids bind to the shallow, surface-exposed cavities of albumins with low affinity and in rapid exchange with the solution, consistent with the physiological role of these allergens as transport proteins^36–38^. In the case of the group 5 mite allergens, crystal studies of Der p 5 and its homologue Der f 21 reveal a helical bundle which can assemble into a dimeric structure, the interface of which encloses a 3000 Å³ cavity^39,40^. While the size of this cavity is comparable to that of Bla g 1, docking studies along with crystal structures of the related allergen Der f 21 reveal an average occupancy of only a single fatty-acid or PEG detergent molecule^39,41^. Moreover, subsequent studies suggest that Der p 5 exists predominantly in the monomeric form, in line with fluorescence assays which reveals an average of 0.66 fatty acid ligands per Der p 5^41^. Given the immunomodulatory abilities of lipids and other hydrophobic ligands (reviewed by Bublin *et al*. in 2014, among others)^11^, the extraordinary binding capacity of Bla g 1 might allow for the more effective codelivery of potentially adjuventising compounds relative to other lipid-binding allergens, allowing it to exert a disproportionately large impact on the sensitization process.

In addition to the innate immunomodulatory properties of these ligands phospholipids vesicles and other hydrophobic ligands can also influence sensitization through modulating protein stability. In previous studies, this effect was primarily manifested through an increased resistance to gastric digestion, and extended the length of time for which allergens can persist in the environment, and thus the likelihood of immune exposure^42,43^. While this is an important contributor to immunogenicity, our study of Bla g 1 suggests a role for fold stability in endosomal processing. This finding mirrors observations reported by Angelina *et al*. (2016) in which the food allergens Sin a 2 and Ara h 1 were found to interact with phospholipid vesicles in a manner that enhanced their resistance to endosomal degradation^19^. However, the structural basis for this interaction and the resulting proteolytic resistance was not discussed. It should be noted that the previous study made no attempt to remove excess lipid. This fact, combined with the lack of a defined lipid binding cavities suggests that both globulin proteins associate with the bilayer surface in a non-specific manner, reducing the exposed surface area available for proteolysis, as has been observed previously for the apple allergen Mal d 1^44^. The nonspecific nature of these interactions potentially raises questions concerning their physiological relevance; indeed, the stability of Ara h 1 showed little enhancement in the presence of lipids from its natural allergen source. In contrast, the studies presented herein focus on the ability of an inhaled allergen (Bla g 1) to bind lipid cargoes in a specific manner via an internal binding cavity, and employ high-resolution structural data and MD studies to demonstrate the ability of these ligands to significantly alter the intrinsic conformational stability of the protein fold on the molecular level without the need for large bilayer structures. Additionally, our study focuses on ligands which are known to be retained by Bla g 1 from the allergen source material (nMix), providing insight into the determinants of Bla g 1 allergenicity within the context of the human exposome.

Reducing the rate at which allergens are cleaved by cathepsin S has been proposed to skew the immune system towards a T_H_2 response by preventing premature endosomal degradation and effective MHCII loading^45–48^. This phenomenon likely contributes to the hypoallergenic nature of certain Bet v 1 isoforms possessing reduced fold stability, and has even been used in the design of new immunotherapies for a range of allergens such as Fel d 1, Bet v 1, and Der p 2 which aimed to enhance MHCII loading^49,50^. While these attempts generally employ engineered versions of the targeted allergen, the observations presented in this study raise the possibility that Bla g 1 or other lipid binding allergens such as Bet v 1 or Der p 2 in either its apo form or loaded with specialized ligands could have similar therapeutic applications without the need for extensive protein modification. This ability of allergen source-derived lipids to influence cathepsin proteolysis rates also raises the interesting possibility that hydrophobic ligands could differentially alter the accessibility of specific cut sites, giving rise to different processing pathways with altered T-cell epitope sequences and distributions. SDS-PAGE analysis of the cleavage of apo and cargo-loaded Bla g 1 by Cat S suggested qualitative differences in both the identity of the fragments generated and their relative populations. Recent solution-NMR and molecular dynamics simulations carried out on various Bet v 1 isoforms suggest that enhanced μs-ms-timescale flexibility at specific regions facilitate the transient exposure of cathepsin S cut sites for proteolysis, giving rise to the different proteolytic cleavage patterns^22^. Ligand binding has the potential to induce similar changes to Bla g 1; indeed, our MALDI experiments identified 6 fragments which are present in the apo digestion reaction, but not its nMix-loaded (^15^N) counterpart, potentially reflecting the presence of cryptic cut sites which are only accessible in the apo form (Supplementary Table S5). Similarly, the perturbations observed in helix 6/6’ upon binding phospholipid, but not fatty-acid ligands could similarly reveal cathepsin sites, resulting in fragmentation patterns that reflects not only the presence of hydrophobic ligands, but also the identity of these ligands and their ability to fit within the Bla g 1 cavity with minimal perturbation. Since it has been previously demonstrated that T_H_1/T_H_2 differentiation is influenced by both the amount of epitopes generated for a given antigen and the affinity of these epitopes for MHCII, the ability of a single protein sequence to give rise to different epitope sequences depending on the context in which it is presented has important implications for the design of allergy treatments^46^.

In conclusion, this paper has characterized the lipid binding properties of Bla g 1; a novel fold whose unique structure supports an unprecedented binding capacity, fulfilment of which produces significant changes in its thermodynamic properties. These changes and their concomitant effect on simulated endosomal processing bear similarities with previous studies, with potential implications for allergencity. Future *in vivo* studies will be required to verify this allergenic hypothesis in the context of Bla g 1.

## Experimental Procedures

### Bla g 1 purification and cargo loading

Bla g 1 in the paper refers specifically to Bla g 1.0101 (World Health Organization, International Union of Immunology Society Subcommittee on Allergen Nomenclature: www.allergen.org, and GenBank accession no. AF072219) residues 34–216. Bla g 1 was expressed in BL21 *E. coli* as a fusion with Glutathione-S-Transferase (GST). Cells were grown in 2x YT media to an OD of ~0.8 at 37 °C. Expression was induced with 0.5 mM IPTG at 16 °C overnight. To produce samples for ^19^F-NMR, cells were grown overnight in 1L LB media, harvested, and subsequently transferred to M9 media supplemented with 50 mg/L L-tryptophan, 50 mg/L-tyrosine, 70 mg/L D/L-3-fluorophenylalanine, and 1 g/L glyphosate (N-(phosphonomethyl)glycine) 1 hr prior to induction^51^. To produce uniformly ^15^N-labeled Bla g 1 for ^15^N-NMR, cells were instead transferred to M9 media with ^15^NH_4_Cl as the sole nitrogen source. Bla g 1 was purified from the resulting cells using an immobilized glutathione column, and the GST tag was subsequently removed as described previously^6^. The resulting protein was then loaded onto a C18 column equilibrated with 0.1% Trifluoroacetic Acid (TFA) in H_2_O. Bla g 1 was eluted using a linear gradient from 0–95% organic buffer (0.1% TFA in acetonitrile). The resulting Apo-Bla g 1 was collected and lyophilized.

Lyophilized Apo-Bla g 1was resuspended in refolding buffer (25 mM HEPES pH 7.5, 75 mM NaCl, 4% DMSO), heated to 95 °C in a hot water bath, and allowed to gradually cool to room temperature over the course of 60–90 minutes. Samples were then buffer exchanged into the relevant buffer (PBS pH 7.5 or 100 mM citrate pH 5.4) using a centrifugal filter (Amicon) to obtain the final Apo-Bla g 1 product. Cargo-loaded Bla g 1 was produced by carrying out the annealing process in the presence of either a >16x or >8x molar excess of the desired fatty acid/diacyl chain ligand respectively. A 1:1:1 molar ratio of palmitate, oleate, and stearate (nMix) was used to replicate the endogenously-bound cargo previously identified in Bla g 1 purified from its native source material^6^. Protein concentration was determined using a BCA assay (Pierce). Endotoxin levels were determined using a chromogenic *Limulus* Amebocyte Lysate assay (Pierce). Heat-inactivated laminarin was added to the provided LAL reagent at a final concentration of 20 μg/mL to prevent activation by β-glucan^52^.

### Computational studies

Using molecular dynamics, solution structures of the Bla g1 peptide and its lipid- and phospholipid-bound forms were generated. The initial structure of Bla g1 for simulations was taken from the X-ray crystal structure from the PDB code 4JRB. Lipids and phospholipids were manually introduced and energy-minimized using the program Amber, version 16^53^. The lipid complexes and a ligand-free Bla g1 system were solvated in separate boxes of water (23133 water molecules in the lipid or phospholipid systems and 23483 in the lipid-free system). Prior to equilibration, all systems were subjected to (1) 100-ps belly dynamics runs with fixed peptide, (2) minimization, (3) low temperature constant pressure dynamics at fixed protein to assure a reasonable starting density, (4) minimization, (5) step-wise slow heating molecular dynamics at constant volume, and (6) constant volume unconstrained molecular dynamics for 10 ns. All final unconstrained trajectories were calculated at 300 K under constant pressure (110 ns, time step 1 fs) using the PMEMD module of Amber.16 to accommodate long range interactions^53^. The parameters were taken from the FF14SB force field for the protein and the lipid and phospholipid parameters were from lipid14 in the AMBER.16 package. Partial charges on lipids were calculated using Gaussian.09^54^ at the B3LYP/6-31 g level.

### Circular dichroism

Circular dichroism spectra were collected on 0.5 μM Bla g 1 in CD buffer (100 mM Tris pH 7.5, 50 mM NaCl) at 25 °C using a Jasco J-815 CD spectropolarimeter. Each spectrum represents the average of four accumulations scanning at 20 nm/min with a data-pitch of 0.2 nm. Each spectrum was acquired on at least three independent trials representing at least two biological replicates. To measure thermostability, samples were heated from 25 °C to 95 °C at a rate of 0.5 °C/min., and the ellipticity at 222 nm was monitored. The data was fit using a two-state Boltzmann curve to determine the melting temperature (T_m_) at α-helical content was reduced by 25%.

### NMR

^1^H-^15^N-NMR experiments were carried out using an 800 MHz 5 mm HCN triple-resonance cryogenically cooled probe on an Agilent DD2 NMR spectrometer. Samples were prepared at ~50–100 μM and maintained at 37 °C. 2D ^1^H-^15^N HSQC spectra were collected at 37 °C with 32–192 scans, with 128 increments in the indirect dimension. The resulting spectra were processed using NMRPipe^55^ and NMRViewJ^56^. Spectra were normalized for both protein concentration and number of scans with the exception of Apo-Bla g 1 due to the poor signal to noise resulting from its unfavourable dynamics timescale. ^19^F-NMR experiments were carried out using an Agilent DD2 600 MHz spectrometer equipped with a 5 mm HCF triple resonance Z gradient probe. Sample were prepared at ~50–200 μM and maintained at 25 °C. 1D ^19^F-NMR spectra were collected with 24576–40690 scans at 25 °C. ^31^P-NMR experiments were carried out on the same spectrometer and temperature using a Varian broadband 5 mm Z-gradient probe. Standard samples of DSPC were prepared in pH 8.0 PBS, and diluted 50% with cholate buffer (100 mM Tris pH 8.0, 100 mM NaCl, 10% w/v cholate) prior to use. Spectra were collected over 1024 scans at 25 °C, and analysed using NMRPipe and NMRViewJ to obtain peak intensities, which were plotted to produce a linear standard curve. Samples of Bla g 1 were loaded with DSPC and buffer exchanged into PBS (pH 8.0) and diluted 50% with cholate buffer to solubilize the Bla g 1-bound DSPC. The intensity of the DSPC ^31^P-NMR peak was compared to the standard curve and protein concentration to obtain an estimate of the binding stoichiometry. Reported values and error bars represent the average and standard deviation obtained from three independent trials representing two biological replicates.

### Cathepsin proteolysis

5–75 µM Bla g 1 was incubated with 0.25 U cathepsin S (EMD Millipore) in protease buffer (100 mM citrate pH 5.4, 2 mM DTT) at 37 °C as described by Machado *et al*.^23^. Samples were removed after 30 and 60 minutes, and analysed using SDS-PAGE. Gels were stained using Coomassie blue, and the intensity of the product and substrate band(s) was quantified using ImageJ^57^. Proteolysis rates were found to increased linearly with Bla g 1 concentration across the ranges tested. As such, rates were recorded relative to Bla g 1 concentration, and normalized to the equivalent values obtained for Apo-Bla g 1 to obtain a relative rate of proteolysis.

### Mass spec analysis

20 μM Bla g 1 was incubated with 0.25 U cathepsin S in protease buffer as described previously. Samples were removed at various timepoints and quenched with 1% TFA. Fragments were identified by both LC-ESI-MS and MALDI-MS. For LC-ESI-MS, 1 µg of digest was analysed on a Q Exactive Plus mass spectrometer (ThermoFisher Scientific) interfaced with a M-Class nanoAcquity UPLC system (Waters Corporation) equipped with a 75 µm × 150 mm BEH dC18 column (1.8 µm particle, Waters Corporation) and a C18 trapping column (180 µm × 20 mm) with 5 µm particle size at a flow rate of 400 nL/min. The trapping column was positioned in-line of the analytical column and upstream of a micro-tee union which was used both as a vent for trapping and as a liquid junction. Trapping was performed using the initial solvent composition. Peptides were eluted by using a linear gradient from 99% solvent A (0.1% formic acid in water (v/v)) and 1% solvent B (0.1% formic acid in acetonitrile (v/v)) to 40% solvent B over 60 minutes. For the mass spectrometry, a data acquisition method was employed with an exclusion time of 15 seconds and an exclusion of +1 charge states. The mass spectrometer was equipped with a nanoflex source and was used in the positive ion mode. Instrument parameters were as follows: sheath gas, 0; auxiliary gas, 0; sweep gas, 0; spray voltage, 2.7 kV; capillary temperature, 275 °C; S-lens, 60; scan range (m/z) of 200 to 2000; 2 m/z isolation window; resolution: 70,000; automated gain control (AGC), 2 × 10e5 ions; and a maximum IT of 200 ms. Mass calibration was performed before data acquisition using the Pierce LTQ Velos Positive Ion Calibration mixture (ThermoFisher Scientific). For quantitative mass spec, 20 μM samples of nMix-loaded, uniformly ^15^N-labeled Bla g 1 and Apo natural abundance (NA) Bla g 1 were incubated separately with 0.25 U cathepsin S in protease buffer at 37 °C. Equal volume samples were removed from both reactions at fixed timepoints, pooled, and quenched by the addition of 1% TFA as described previously. The pooled ^15^N/^14^N samples were analysed by MALDI-MS to obtain peak intensities and thus relative populations for the various cleavage fragments. Briefly, samples were desalted using C18 ZipTips (EMD-Millipore) using essentially the manufacturer’s recommended protocol. Desalted peptides were spotted onto a stainless steel target and mixed with 33% saturated α-cyano-4-hydroxycinnamic acid in 1:1 (0.2% formic acid:acetonitrile) and analysed using an Applied Biosystems 4800 ToF mass spectrometer in the reflector mode. Data were collected from m/z 800 to m/z 4000 with a focus mass of m/z 1700 and accumulation of 2000 subspectra.

## Supplementary information

Supplementary Information

## Data Availability

All thermostability and cathepsin S kinetics data generated or analysed during the current study are included in its Supplementary Information Files. All other datasets generated or analysed during the study are available from the corresponding author on reasonable request.
