# Supplementary material for: Hydrophobic ligands influence the structure, stability, and processing of the major cockroach allergen Bla g 1
Source: Sci Rep. 2019 Dec 4;9:18294. doi: 10.1038/s41598-019-54689-8 (PMC6893020; doi:10.1038/s41598-019-54689-8)
Supplement: Supplementary file 1 — Supplementary Information [file 41598_2019_54689_MOESM1_ESM.pdf]

Hydrophobic ligands influence the structure, stability, and processing of the major cockroach allergen Bla g 1

Supplementary Information

Alexander C.Y. Foo[1†], Peter M. Thompson[1†], Lalith Perera[1], Simrat Arora[1], Eugene F. DeRose[1], Jason Williams[2], and Geoffrey A. Mueller[1*]

From the [1]Genome Integrity and Structural Biology Laboratory, National Institute of Environmental Health Sciences, NIH, HHS, Research Triangle Park, NC 27709; [2] Mass Spectrometry Research and Support Group, National Institute of Environmental Health Sciences, NIH, HHS, Research Triangle Park, NC 27709

[*] To whom correspondence should be addressed: Geoffrey A. Mueller: [1]Genome Integrity and Structural Biology Laboratory, National Institute of Environmental Health Sciences, Research Triangle Park, NC. 27709; Mueller3@nih.gov; Tel: (984) 287-3589
[†] Both authors contributed evenly to the work presented herein

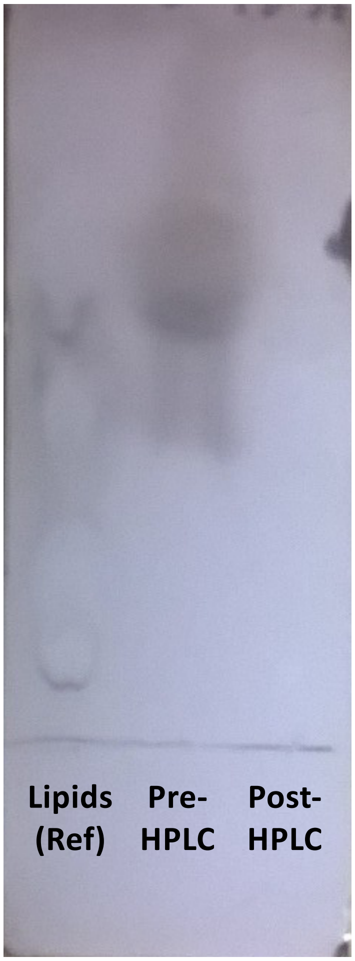

S1: Thin-Layer Chromatography (TLC) plate illustrating the removal of bound lipids from Bla g 1 via C18 HPLC purification

S2a: Melting temperatures (T_m) of Bla g 1 bound to various hydrophobic cargoes as shown in Fig. 4.

**Cargo**	**T_m (^oC)**
Apo	43±4
Laurate (C12)	44±3
Myristate (C14)	46±1
Palmitate (C16)	63±6
nMix (C16-18)	62±4
nBla g 1	66±2
Stearate (C18)	68±2
Arachidate (C20)	66±5
Behenate (C22)	56
POPC (16:0-18:1 PC)	58±13
DOPC (18:1 PC)	67±6
DSPC (18:0 PC)	74±5
POPE (16:0-18:1 PE)	66±9
DOPE (18:1 PE)	52
DSPE (18:0 PE)	72±4
POPG (16:0-18:1 PG)	75
LTA (S. aur)	65±7
LTA (B. sub)	67±1
Lipid A	52±3

S2b: Relative cathepsin S proteolysis rates of Bla g 1 bound to various hydrophobic cargoes as shown in Fig. 5.

**Cargo**	**Rel. Rate of Proteolysis**
Apo	1.0±0.2
Laurate (C12)	0.7±0.1
Myristate (C14)	0.4±0.1
Palmitate (C16)	0.46±0.06
nMix (C16-18)	0.2±0.1
Stearate (C18)	0.12±0.05
Arachidate (C20)	0.31±0.08
Behenate (C22)	0.02±0.01
POPC (16:0-18:1 PC)	0.5±0.1
DOPC (18:1 PC)	0.7±0.1
DSPC (18:0 PC)	0.7±0.2
POPE (16:0-18:1 PE)	0.5±0.1
DOPE (18:1 PE)	0.8±0.1
DSPE (18:0 PE)	0.77±0.07
POPG (16:0-18:1 PG)	0.37±0.04
LTA (S. aur)	0.5±0.1
LTA (B. sub)	0.8±0.2

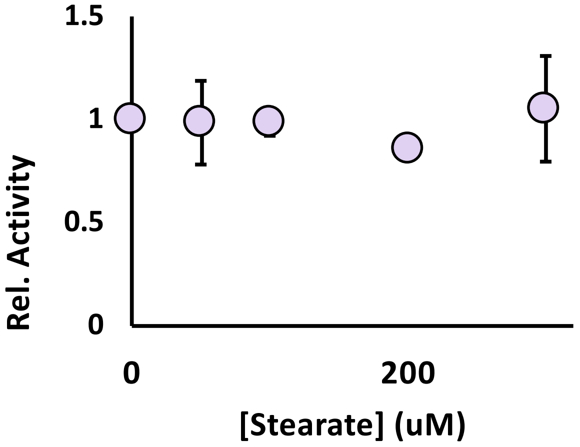

S3: Effect of Stearate Micelles on Cathepsin S Activity. Rate of Bla g 1 proteolysis by Cathepsin S in the presence of stearic acid micelles. Stearic acid was added to Apo-Bla g 1 after annealing. Excess stearate was not removed prior to proteolysis. Reactions carried out using 30 uM Bla g 1, and the resulting rates are normalized against the rate observed in the absence of stearate (0 uM)

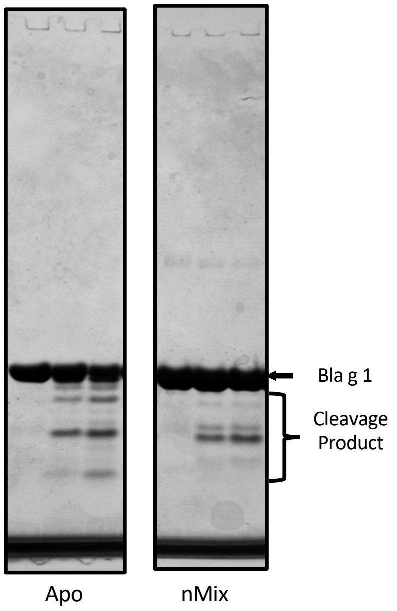

S4: Assessing Bla g 1 proteolysis via SDS-PAGE. Full-length view of the gels shown in Fig. S5.

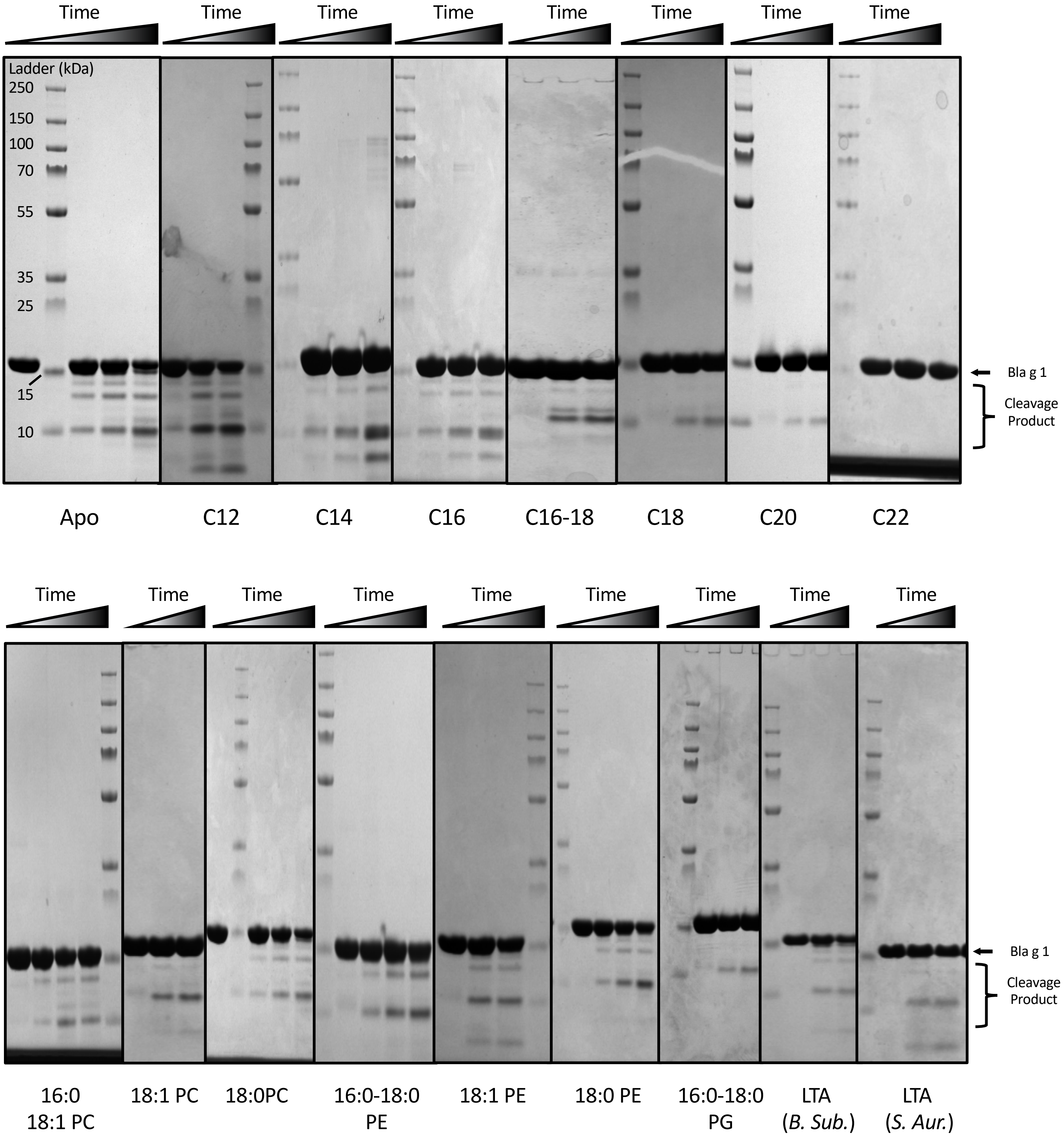

S5: Assessing Bla g 1 proteolysis via SDS-PAGE. Representative SDS-PAGE gels used to generate the data shown in figure 5. Note that each column in figure 5 represents data obtained from at least three trials encompassing at least two biological replicates.

S5: Identity of all the Bla g 1 peptide fragments identified in the quantitative MALDI mass spec, along with the ratio of peak intensities for the ^{14}N and ^{15}N isotope peaks. Peaks without a corresponding ^{15}N isotope peak are indicated with *

Start Residue	Sequence	M/z (^{14}N+ ^{1}H)	M/z (^{15}N+ ^{1}H)	^{15}N:^{14}N Ratio
122	(A)ILPIEDLK(A)	940.574	949.545	*
148	(N)AIRSPEFQ(S)	947.495	959.459	0.117
23	(L)IPVDQIIAI(A)	981.598	991.568	0.116
121	(L)AILPIEDLK(A)	1011.609	1021.579	0.203
151	(R)SPEFQSIVQ(T)	1034.516	1045.483	*
147	(Y)NAIRSPEFQ(S)	1061.538	1075.496	0.216
176	(R)EKGVDVDKIIE(L)	1244.674	1257.635	*
101	(S)GRKYHIRRGVG(I)	1298.756	1321.687	0.050
145	(A)LYNAIRSPEFQ(S)	1337.685	1353.637	0.111
81	(N)AIDFLNGIHDLL(G)	1340.721	1355.676	0.220
148	(N)AIRSPEFQSIVQ(T)	1374.738	1391.687	0.171
81	(N)AIDFLNGIHDLLG(I)	1397.743	1413.695	0.310
144	(L)ALYNAIRSPEFQ(S)	1408.723	1425.672	0.572
163	(N)AMPEYQNLLQKLR(E)	1603.863	1623.803	0.0370
176	(R)EKGVDVDKIIELIR(A)	1626.943	1645.886	0.062
94	(G)IPHIPVSGRKYHIR(R)	1672.976	1697.901	0.130
148	(N)AIRSPEFQSIVQTLN(A)	1702.913	1723.85	0.074
93	(L)GIPHIPVSGRKYHIR(R)	1729.998	1755.92	0.051
76	(E)ANGLNAIDFLNGIHDLLG(I)	1866.971	1889.902	*
176	(R)EKGVDVDKIIELIRALF(-)	1958.133	1980.067	0.150
94	(G)IPHIPVSGRKYHIRRGVG(I)	2042.189	2074.093	0.023
145	(A)LYNAIRSPEFQSIVQTLN(A)	2093.103	2118.028	0.066
93	(L)GIPHIPVSGRKYHIRRGVG(I)	2099.21	2132.111	0.015
173	(Q)KLREKGVDVDKIIELIRALF(-)	2355.413	2384.326	*
172	(L)QKLREKGVDVDKIIELIRALF(-)	2483.471	2514.378	*
163	(N)AMPEYQNLLQKLREKGVDVDKIIELIRALF(-)	3542.977	3584.851	0.134
